# Monoclonal-Antibody-Based Immunoassays for the Mycotoxins NX-2 and NX-3 in Wheat

**DOI:** 10.3390/toxins16050231

**Published:** 2024-05-18

**Authors:** Chris M. Maragos, Martha M. Vaughan, Susan P. McCormick

**Affiliations:** Mycotoxin Prevention and Applied Microbiology Research Unit, National Center for Agricultural Utilization Research, Agricultural Research Service, United States Department of Agriculture, 1815 N University, Peoria, IL 61604, USA; martha.vaughan@usda.gov (M.M.V.); susan.mccormick@usda.gov (S.P.M.)

**Keywords:** antibody, immunoassay, NX toxins, trichothecenes, deoxynivalenol, wheat

## Abstract

The fungal infestation of crops can cause major economic losses. Toxins produced by the causative fungi (mycotoxins) represent a potential safety hazard to people and livestock consuming them. One such mycotoxin is deoxynivalenol (DON, also known as vomitoxin), a trichothecene associated with Fusarium Head Blight of wheat. DON is commonly found in cereal crops worldwide. A group of trichothecene mycotoxins closely related to DON, the NX toxins, have been reported to occur in the northeastern United States and southern Canada. While many commercial immunoassays are available to detect DON, there are no rapid screening assays for the NX toxins. We describe the development and isolation of three monoclonal antibodies (mAbs) specific towards two NX toxins: NX-2 and NX-3. The mAbs did not recognize DON or several other closely related trichothecenes. One of the mAbs was selected for development of an enzyme-linked immunosorbent assay (ELISA) for NX-2 and NX-3 in wheat. The dynamic ranges for the assay were 7.7 to 127 μg/kg for NX-2 and 59 μg/kg to 1540 μg/kg for NX-3 in wheat. Recoveries from spiked wheat averaged 84.4% for NX-2 and 99.3% for NX-3, with RSDs of 10.4% and 11.3%, respectively (n = 24). The results suggest that this assay can be used to screen for NX toxins in wheat at levels relevant to human food and animal feed safety.

## 1. Introduction

Fusarium Head Blight (FHB) is among the diseases that cause the greatest disruption to wheat and barley production worldwide [1,2]. The disease is caused by members of a group of fungi known generally as the *Fusarium graminearum* species complex. The fungi within this species complex contaminate a wide variety of cereal crops including wheat, barley, and maize. During the process of infesting plants, the fungi may produce virulence factors to weaken the plant’s defenses and facilitate infection [3]. They can also produce low-molecular-weight secondary metabolites that are toxic. These may include compounds toxic to microorganisms (antibiotics), and/or animals (mycotoxins). These classifications are not mutually exclusive, as certain mycotoxins are known to act as virulence factors and have antibiotic effects. Among the latter are a structurally diverse group of mycotoxins based upon a trichothecene core [4].

**Figure 1:**
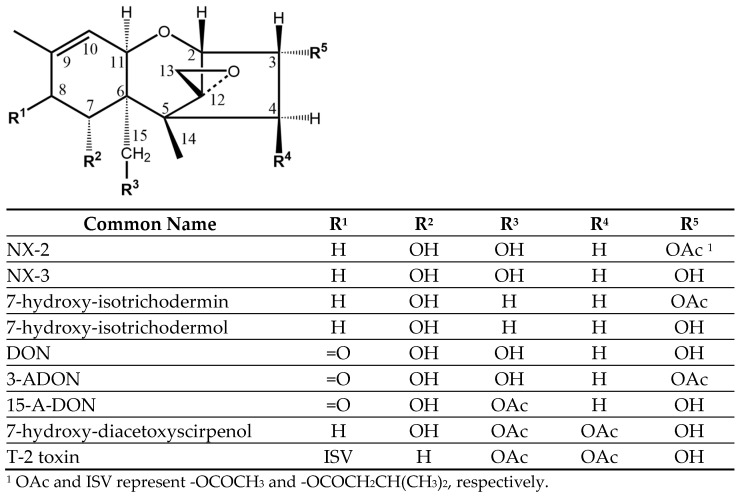
Structures of some of the trichothecene mycotoxins.

There are several chemical classification systems for the trichothecenes. One commonly used system is that of Ueno [5], who created four groups (A-D) based upon the substituents to the trichothecene skeleton (Figure 1). The archetype of the group A trichothecenes, which contain an oxygen function other than a ketone at position C-8 (or no oxygen there at all), is a T-2 toxin. Deoxynivalenol (DON) is the archetype of group B trichothecenes. Members of this group contain a carbonyl at C-8. DON, which can be produced by many isolates of *F. graminearum*, is a virulence factor and one of the most widely reported toxins associated with FHB. The NX toxins are a more recently discovered series of trichothecenes. NX-3 is identical to DON except at C-8 (R1 in Figure 1), where it lacks the carbonyl present in DON. NX-2 is an acetylated form of NX-3. Because the NX toxins lack a carbonyl at C-8, they are classified as group A trichothecenes, a distinction that may be important for their toxicity.

Toxicity of trichothecenes is related to the presence of the C12-C13 epoxide and the C9-C10 double bond [6,7]. Many of the trichothecenes disrupt protein synthesis in eukaryotes by binding to the peptidyl transferase site of ribosomes. DON induces ribotoxic stress by activating mitogen-activated protein kinases (MAPKs) [8]. Symptoms vary depending upon the animal species and the dose. DON is known colloquially as “vomitoxin” because of its ability to induce emesis in pigs and has been associated with gastroenteritis in humans [9,10]. The chronic ingestion of trichothecenes at low doses can lead to anorexia, the retardation of growth, immunotoxicity, impaired reproduction, and impaired development [9]. The range of affected species is broad, including swine, rats, mice, poultry, and fish [8]. The T-2 toxin is often considered as intrinsically more toxic than DON and symptoms have some overlap with those observed with DON. Symptoms include emesis, feed refusal, stomach necrosis, and dermatitis [11]. In humans, the T-2 toxin has been associated with the gastrointestinal disorder alimentary toxic aleukia [12].

Having been discovered more recently relative to DON and the T-2 toxin [13], there are fewer toxicological studies of the NX series of toxins. Results from two in vitro translation assays indicated that NX-3 inhibited protein synthesis equivalently to DON, while NX-2 was less toxic [13]. NX-3 has also been shown to trigger enhancement in intracellular reactive oxygen species (ROS) in human liver (HepG2) cells [14]. In addition, NX-3 was found to be equipotent to DON in activating the pro-inflammatory effects of the nuclear factor kappa B (NF-kB) signaling pathway [14]. In human intestinal Caco-2 cells, NX-3 exhibited similar cytotoxicity to DON, while NX-2 was less toxic [15].

Given the worldwide distribution of the *F. graminearum* species complex, and the widespread occurrence of Fusarium Head Blight, cereal crops are routinely found to be contaminated with trichothecenes, in particular DON and its acetylated derivatives (3-ADON, 15-ADON; Figure 1). The biosynthetic machinery involved in trichothecene production is diverse, with the diversity presumably derived from both the widespread geographical distribution of the species and the variety of hosts that can be infested [16,17,18]. This genetic diversity is the basis for the wide variety of trichothecene structures found [19]. The patterns of secondary metabolites that are produced are commonly used to classify the *F. graminearum* species complex into chemotypes [20]. There are three commonly accepted chemotypes: those that produce primarily DON and 15-ADON, those that produce primarily DON and 3-ADON, and those that produce primarily nivalenol (NIV) [21]. The populations that produce NX toxins represent a more recently described, fourth, chemotype. Currently, the NX toxins have only been found to be produced by certain isolates of *F. graminearum*, and possibly *F. culmorum* [22]. Isolates of *F. graminearum* producing NX-2 and NX-3 have, so far, been found predominantly in the northern United States and southern Canada and infest wheat, maize, barley, and oats [21,23,24,25,26]. It has been suggested that the NX chemotype may represent as much as 20% of the *F. graminearum* population in parts of the state of New York [26]. Most of the scientific literature refers to the NX toxins using the nomenclature described by the discoverers of the group [13]. In some cases, a different nomenclature, more consistent with that developed for DON, has been used [27]. In the latter case, “3ANX” is equivalent to NX-2 and “NX” is equivalent to “NX-3”. While logical, in this report, we use the nomenclature established by Varga et al. (Figure 1) as this nomenclature dominates the literature.

The NX toxins were discovered during efforts to produce atoxigenic biocontrol strains of *F. graminearum* [13]. Strains were found that, by genotype, were expected to be 3-ADON producers, but which, when grown on rice, did not produce acetylated DON, DON, or NIV as determined by HPLC-MS/MS [28]. A combination of high-resolution MS (HRMS) and HR tandem MS (HRMS/MS) was used to help identify the new trichothecenes, which were termed NX toxins. The same group also used GC-MS to detect the trimethylsilyl (TMS) derivative of NX-3 and NX-2 in inoculated wheat heads. Among the analytical methods for measuring NX toxins, those using LC-MS/MS [22,27,29,30,31] and GC-MS of the TMS derivative [24,32] predominate. NX-2 and NX-3 have been reported to have a UV absorption maximum at 196 nm with a logƐ of 3.81 in ethanol [27]. There is a single report where the usefulness of commercially available DON immunoassays for the detection of NX toxins was evaluated [33]. Six commercial immunoassays and two previously developed DON monoclonal antibodies (mAbs) were tested. Of these, only one assay, using one of the non-commercial immunoassays, cross-reacted significantly with NX-3 at 14% on a molar basis. None of the immunoassays cross-reacted with NX-2 [33]. The analytical methods developed to detect the NX toxins have generally been applied to measuring the toxins in either field inoculated samples or in cultures of strains isolated from cereal grains. The authors are unaware of any surveys for the NX toxins in commodities such as wheat, barley, or maize, perhaps because of a lack of tools for rapid screening for these toxins. 

Because of the toxicity and potential occurrence in commodities, particularly in wheat and barley from the northeastern US and southern Canada, a rapid immunoassay for screening for NX toxins is needed. Our objectives were to produce monoclonal antibodies with good recognition for NX-2 and NX-3 and then to apply the antibodies as components of immunoassays for the rapid detection of these emerging mycotoxins in wheat.

## 2. Results

### 2.1. Production of NX Antibodies

Two immunogens, NX3-OVA and NX3-BSA, were each administered to 10 mice and the resulting antisera were evaluated by an enzyme-linked immunosorbent assay (ELISA). The format involved using the orthogonal conjugate as the immobilized antigen (e.g., NX3-BSA for the NX3-OVA sera and vice versa). In this assay format, competition was between the immobilized antigen and free toxin (NX-3 or NX-2) for binding to antibodies present in the sera. Responses from mice immunized with NX3-BSA were quite good. The first set of antisera from all 10 mice, collected six weeks following the primary immunization, were clearly inhibited by 1 μg/mL of NX-3, a level of toxin at which one of our current DON monoclonal antibodies (mAb) also responded [33]. Furthermore, the response to NX-3 improved with the second set of sera from this group. Again, all 10 mice responded to NX-3; however, 5 of them were inhibited 50% or more by 100 ng/mL of NX-3. For the second group of mice, those immunized with NX3-OVA, 4 of the 10 responded to 100 ng/mL when evaluated at the first bleed and 7 of the 10 responded to this same level of NX-3 at the second bleed. Before proceeding to splenocyte fusions, the three best sera from each group of mice were compared. The best mouse from the NX3-BSA group responded with 50% inhibition (IC_50_) at circa 20 ng/mL of NX-3, while the best mouse from the NX3-OVA group demonstrated an IC_50_ of circa 10 ng/mL. Given the slightly better response, the best mouse from the NX3-OVA group was selected for splenocyte fusion and hybridoma production. Fifteen stable hybridomas were isolated from the splenocyte fusion, ten of which were considered sensitive enough to NX-3 to pursue cloning. One of the ten lost the ability to produce the antibody during cloning. Cells from the remaining nine were administered to mice, yielding ascites fluid for evaluation. Of the nine, the three with the greatest activity towards NX-3 were selected for further evaluation. These three were cell lines 1-4.2.7, 1-8.1.7, and 1-13.2.1, hereafter referred to as 1-4, 1-8, and 1-13, respectively.

### 2.2. Effects of Methanol

The three NX antibodies were evaluated for their ability to perform in buffer mixtures containing methanol (MeOH). Calibration curves of NX-2 were prepared in a simple PBS buffer, and a PBS buffer containing up to 30% MeOH. Results in Table 1 are presented two ways: as the concentration of NX-2 required to inhibit color development in the assay by 50% (IC_50_) and the relative increase in IC_50_ (i.e., decrease in sensitivity) associated with increasing MeOH concentrations. The effect of MeOH on the signal values in the absence of any analyte is provided in Appendix A (Table A1).

With IC_50_s below 1 ng/mL in PBS, it was clear that all three antibodies demonstrated excellent sensitivity towards NX-2. The three antibodies responded similarly, but not equally, to the presence of increasing MeOH. All three demonstrated an approximate doubling of IC_50_ with 10% MeOH; mAb 1-8 was slightly less affected by MeOH, as evidenced by the slightly better relative effects at all three MeOH concentrations. The difference was most evident at the highest MeOH concentration.

### 2.3. Cross-Reactivity with Structural Analogs

While the NX toxins are classified as group A trichothecenes, their structures (Figure 1) are very close to those of DON and its acetylated analogs. The ability of the three NX mAbs to recognize structural analogs was evaluated by ELISA in the presence of 10% MeOH/PBS (Table 2). 

Data in Table 2 are presented in the form of sensitivity towards the analog (IC_50_) and in the form of how they cross-reacted relative to the NX-2 toxin. The percentage of the cross-reaction was determined by dividing the IC_50_ of the antibody for NX-2 by the IC_50_ of the antibody for the analog. Results indicated that all three of the antibodies were highly specific for NX-2, with a small amount of the cross-reaction to NX-3, a slight cross-reaction to 3-ADON, and very little, or no, cross-reaction with DON, 15-ADON, 7-hydroxy isotrichodermol, 7-OH isotrichodermin, or the T-2 toxin. Of the three antibodies, mAbs 1-8 and 1-13 were similar in cross-reactivity towards NX-3 and cross-reacted slightly higher with this toxin than mAb 1-4.

### 2.4. Application to Spiked Wheat

**Figure 2:**
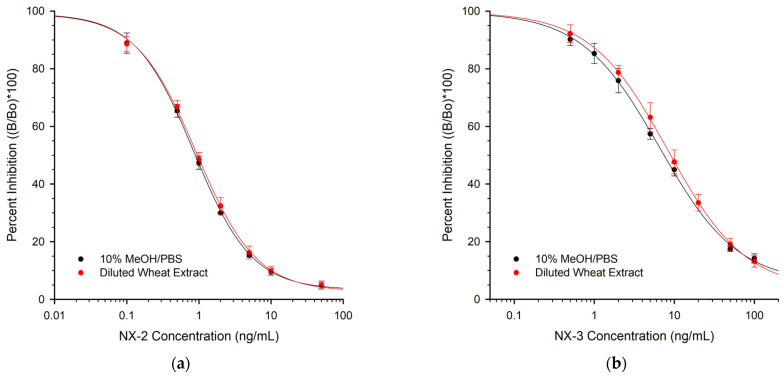
The effect of the wheat matrix on the response of the mAb 1-8 ELISA: (**a**) NX-2; (**b**) NX-3. Each line is the average obtained from three plates. Error bars are ± 1 standard deviation. Average absorbance values for the toxin-free samples in (**a**) were 0.953 and 0.985 (solvent, diluted wheat extract, respectively). Average absorbance values for the toxin-free samples in (**b**) were 1.04 and 1.09 (solvent, diluted wheat extract, respectively).

One of the three mAbs (1-8) was selected for use in developing an assay for NX-2 and NX-3 in wheat. Wheat was extracted with 80% MeOH/H_2_0, then diluted 8-fold with a PBS buffer before testing, resulting in test solutions containing 10% MeOH. To assess the impact of the matrix on the performance of the ELISA, extracts from control wheat (non-detectable NX toxins) were diluted 8-fold and then used as the matrix for the preparation of calibration curves (Figure 2).

Because the diluted extracts contained 10% MeOH, the comparison was made to PBS containing 10% MeOH. The diluted wheat matrix did not significantly affect the shape of the calibration curve for NX-2 (Figure 2a). However, for NX-3, the presence of the matrix shifted the curve slightly to the right (Figure 2b). For immunoassays, the limit of detection (LOD) and limit of quantification (LOQ) can be represented in several ways. It is common for LOD to be estimated as the level that will generate signal inhibition equivalent to three times the standard deviation from the control (blank) sample. The LOQ, considered the lower end of the dynamic range, is often calculated from the concentration that causes a 20% change in the signal (IC_20_). Conversely, the upper end of the dynamic range is the IC_80._ An analysis of these parameters for the mAb-1-8-based ELISA is depicted in Table 3.

From the assay parameters, and the dilution factor used in the assay (0.03125 g equivalents of wheat per mL of diluted extract), it was possible to estimate the dynamic range expected in wheat. For NX-2, this was 7.7 to 127 μg/kg and for NX-3, this was 59 to 1540 μg/kg. In addition to spiked extracts, the recovery of NX-2 and NX-3 from spiked wheat was determined using the mAb 1-8 ELISA. Wheat was spiked with NX-2 or NX-3, at six concentrations with four replicates at each concentration. In each case, the lowest spiking level was selected to be near the anticipated LOQ of the method while the highest spiking level was selected to be near the upper limit of the dynamic range. Results are shown in Table 4 (NX-3) and Table 5 (NX-2).

Recoveries of NX-3 from wheat spiked over the range of 50 to 4000 μg/kg averaged between 93.9% and 105%. The average for all 24 samples spiked with NX-3 was 99.3% with an RSD of 11.3%. Recoveries of NX-2 from wheat spiked over the range of 7.5 to 150 μg/kg averaged between 77.2% and 92.5%. The average for all 24 samples spiked with NX-2 was 84.4% with an RSD of 10.4%. 

## 3. Discussion

The two toxin–protein conjugates, NX3-BSA and NX3-OVA, were relatively similar in their ability to induce an immune response to free NX toxins. The response was sufficiently high that only one splenocyte fusion was needed, from which three mAbs sensitive to NX-2 and NX-3 were eventually isolated. These are not the first antibodies that have been reported to bind with NX-3. That distinction belongs to a DON mAb, which showed cross-reactivity to NX-3 with an IC_50_ of 227 nmol/L, equal to 64 ng/mL [33]. That mAb was also marginally cross-reactive with NX-2, with an IC_50_ of 1340 ng/mL. In comparison, the three mAbs reported here demonstrated IC_50_s of 0.4 to 0.6 ng/mL for NX-2 in PBS (Table 1). We extracted wheat with 80% MeOH and diluted the extracts 8-fold, yielding test solutions containing 10% MeOH. In the presence of 10% MeOH/PBS, the IC_50_s ranged from 0.84 to 1.1 ng/mL (NX-2) and 7.4 to 11.8 ng/mL (NX-3) (Table 2). The better sensitivity that we achieved was likely due to our use of NX–protein conjugates for immunization, compared to DON–protein conjugates used in the previous work [33].

The three NX antibodies had similar cross-reactivity patterns when tested against a panel of trichothecene analogs (Table 2). All three were clearly most sensitive towards NX-2 and less sensitive towards the de-acetylated NX-2 (i.e., NX-3). The location of the acetate in NX-2 is at C-3 (Figure 1). Previous experience with a variety of DON and NIV antibodies has suggested that immunogens made by linking at the C-3 were more sensitive to analogs acetylated at C-3, while those linked at C-15 were more sensitive to analogs acetylated at C-15 [33,34,35,36]. The conjugates used in our immunizations involved NX-3 attached to proteins using a carbodiimide chemistry that could potentially have formed a linkage at one or more of the three hydroxyl groups present in NX-3. The fact that the antibodies demonstrated the greatest sensitivity towards NX-2 suggests that most of the sensitive antibodies were produced by conjugates linked through the C-3 position of the toxin.

Previous studies have indicated that, while the fungi may be producing predominately acetylated derivatives of DON, what is found in tissues of infested plants is typically DON, with the acetylated derivatives representing only 10% to 15% of DON [37]. As with DON, it has been hypothesized that *F. graminearum* produces primarily the acetylated form (NX-2), which is then de-acetylated by the plant or by the environment outside of the fungus to NX-3. In fact, when wheat ears were inoculated with an NX-2-producing strain of *F. graminearum*, the main trichothecene observed in planta was NX-3 [29,38]. At least one mechanism for the conversion of NX-2 to NX-3 may be through the action of plant carboxylesterases [30]. These results suggest that an ELISA for NX toxins would ideally detect the two toxins equally or detect NX-3 better than NX-2. The three mAbs developed here are very sensitive towards NX-2. Fortunately, they are also sufficiently sensitive to NX-3 to be useful in detecting this toxin at relevant levels in wheat.

There are currently no data published on the levels of naturally occurring NX toxins in commodities. However, NX-producing strains have been isolated from important cereal grains [39,40] and inoculation studies have shown that NX-producing strains can contaminate wheat and barley with similar levels of toxin as DON-producing strains [24,38]. As such, a good concentration range to target for NX assays would be a range where DON is relevant. In the United States, the US Food and Drug Administration (USFDA) has established advisory levels for DON in commodities and foods. In finished wheat products for human consumption, this level is 1 ppm (1000 μg/kg). However, levels as high as 30,000 μg/kg are allowable on distillers’ dried grains and related products destined for use in certain ruminant feeds [41]. The ELISAs we have described can detect NX-3 with a dynamic range of 59 to 1540 μg/kg. Higher levels would require a further dilution of the extract (beyond the 1:8 dilution used here) to permit quantification. For NX-2, the ELISA is even more sensitive, with a dynamic range of 7.7 to 127 μg/kg. Furthermore, recoveries from wheat spiked over the range of 50 to 4000 μg/kg of NX-3 or 7.5 to 150 μg/kg were quite good (Table 4 and Table 5). These factors suggest that the assay based upon mAb 1-8 can be used to measure NX-2 and NX-3 toxins at relevant levels in wheat.

## 4. Conclusions

Three sensitive mAbs were developed against the emerging group A trichothecenes NX-2 and NX-3. Indirect competitive ELISAs based on these antibodies performed best in the absence of methanol but could tolerate it at levels up to 30%. All three antibodies were most specific for the NX-2 toxin, with cross-reactivity towards NX-3 of 7 to 12.5%. The application of one of the mAbs (1-8) to spiked wheat revealed that it could be used to detect both NX-2 and NX-3 at concentrations that are anticipated to be found in wheat.

## 5. Materials and Methods

### 5.1. Reagents

DON was obtained from Santa Cruz Biotechnology, Inc. (Dallas, TX, USA). 3-ADON, 15-ADON, the T-2 toxin, o-phenylenediamine (OPD), bovine serum albumin (BSA), chicken egg albumin (OVA), 1,1′-carbonyldiimidazole (CDI), and polyvinyl alcohol (PVA) were purchased from Sigma-Aldrich (Milwaukee, WI, USA). NX-2 was isolated from liquid cultures of *F. graminearum* NRRL44211. NX-3 was prepared by treating NX-2 with 0.1N NaOH. 7-hydroxy isotrichodermin was isolated from cultures of *F. verticillioides* expressing *FgTri1* that were fed isotrichodermin [42]; 7-hydroxy isotrichodermol was prepared by treating 7-hydroxy isotrichodermin with 0.1N NaOH. 7-hydroxy 4,15-diacetoxyscirpenol was isolated from cultures of *F. sporotrichioides* MB1716 [43] transformants expressing Tri1 from *F. graminearum* NRRL29214 [44]. Peroxidase-conjugated goat anti-mouse IgG (GAMP) was purchased from Jackson Immuno Research Laboratories, Inc. (West Grove, PA, USA). Acetonitrile (ACN), methanol (MeOH), and ethanol (EtOH) were HPLC grade and purchased from major suppliers. Phosphate-buffered saline (PBS) was 0.01 M sodium phosphate and 0.145 M sodium chloride, pH 7.2. OVA-PBS was 0.1% (*w*/*v*) OVA in PBS. BSA-PBS was 1% (*w*/*v*) BSA in PBS. Tween–PBS was 0.02% (*v*/*v*) Tween-20 in PBS. PVA-PBS was 1% (*w*/*v*) PVA in PBS, pH 7.2.

### 5.2. Preparation of Stock Solutions and Calibrants

The DON stock solution was prepared at 2 mg/mL in ACN/H_2_O (84 + 16, *v*/*v*) and the concentration of dilutions in ACN was established using the extinction coefficient of 6,805 L/mole*cm [44]. Stock solutions of NX-2, NX-3, 7-hydroxy-isotrichodermol, 7-hydroxy-isotrichodermin, 7-hydroxy-diacetoxyscirpenol, and 7-hydroxy diacetoxyscirpenol were prepared gravimetrically in ACN or ACN/H_2_O (84 + 16). Intermediate dilutions were prepared at 50 μg/mL in H_2_O/ACN (4 + 1 *v*/*v*). For most experiments, the working dilutions of each toxin were prepared daily in a 1 + 9 (*v*/*v*) mixture of MeOH and PBS (10% MeOH/PBS). For NX-2, the calibration standards were generally prepared over the range of concentrations from 0.1 to 50 ng/mL. For NX-3, the range was generally 0.5 to 100 ng/mL. To study the effects of MeOH concentration, NX-2 standards were also prepared in MeOH/PBS at MeOH concentrations of 0% (PBS alone), 20% (1 + 4 *v*/*v*), and 30% (3 + 7 *v*/*v*). Because of the effects of MeOH, for high MeOH concentrations, the upper limit for the calibration standards was increased, to a maximum of 500 ng/mL (in 30% MeOH). Cross-reactivity studies were conducted with trichothecene analogs prepared in 10% MeOH/PBS at concentrations up to 5000 ng/mL.

### 5.3. Preparation and Production of Immunoreagents

NX-3 was conjugated to two proteins, OVA (NX3-OVA) and BSA (NX3-BSA). Each was used to immunize mice, and each was also used as the immobilized antigen in ELISA assays of sera from the opposite group. The conjugates were prepared using a procedure originally developed for DON [34]. Briefly, 6.3 mg (22.3 μmole) of NX-3 was reacted with 110 mg of CDI (678 μmole) in acetone and the mixture reacted with 15 mg of OVA. After reacting for 24 h at 4 °C, the low-molecular-weight reactants and products were removed by dialysis. The conjugate was then lyophilized in 0.5 mg and 1 mg aliquots. In a similar fashion, NX3-BSA was prepared, except substituting 30 mg of BSA for the OVA. Conjugates were stored at −20 °C until used. The products were rehydrated with deionized water as needed. Dried conjugates were sent to Envigo (Madison, WI, USA) for the immunization of mice and collection of antisera. All animal procedures were approved by the Institutional Animal Care and Use Committee (IACUC) of Envigo using protocols developed in accordance with U.S. National Institutes of Health—Office of Laboratory Animal Welfare guidelines. 

Ten female Balb/C mice were given a primary injection of 100 µg of NX3-BSA per animal, using the same procedures as described previously for the production of paxilline antibodies [45]. Similarly, a second group of 10 mice were immunized with NX3-OVA. Immunogens were prepared as 1 + 1.1 (*v*/*v*) mixtures of a conjugate and Freund’s complete adjuvant, with 0.2 mL injected subcutaneously at 4 sites. Mice received a secondary injection of 50 μg in Freund’s incomplete adjuvant after 28 days. Additional booster injections were spaced four weeks apart and consisted of 50 μg of an immunogen in Freund’s incomplete adjuvant. Sera were collected 14 days after boosting and were evaluated by competitive ELISA. 

To screen antisera and culture supernatants, two immobilized antigen ELISAs were developed. One using immobilized NX3-OVA (for screening NX3-BSA antisera) and one using immobilized NX3-BSA (for screening NX3-OVA antisera). These assays are described in Section 5.4. Antisera from each of the 20 mice were evaluated, and the mouse with the best response was selected for splenocyte fusion and hybridoma production. Splenocyte fusions and hybridoma production were conducted at Envigo. Splenocytes from the selected mouse were fused with Balb/C non-immunoglobulin-secreting (NS-1) myeloma cells using polyethylene glycol. The NS-1 cell line was obtained from Envigo. The fused cells were plated in HAT selection media and 11 days later, HAT resistant cultures were isolated. Culture supernatant solutions from the hybridoma culture supernatants were screened using competitive antigen-immobilized ELISA (Section 5.4). 

### 5.4. Immunoassay Procedures

For the evaluation of NX3-BSA antisera, NX3-OVA was initially immobilized at a concentration of 100 ng/mL. For the evaluation of NX3-OVA antisera, NX3-BSA was initially immobilized at a concentration of 20 ng/mL. Immobilization was conducted by incubating 0.1 mL of a conjugate, at the indicated concentration, in a 0.05 M sodium phosphate buffer (pH 7.2), in wells of polystyrene microtiter test plates overnight at 4 °C. The test plates were washed twice with 0.32 mL per well of Tween–PBS, blocked with 0.32 mL per well of PVA-PBS, and incubated at the ambient temperature for 2 h. Test solutions were prepared in the wells of a polypropylene microwell mixing plate (Corning Inc., Corning, NY, USA) and consisted of NX-2 or NX-3 standard solutions mixed with equal volumes of antisera, diluted 1:500 to 1:5000. For screening of hybridoma supernatant solutions, a dilution of 1:10 in OVA-PBS was used. The wells of the test plate, containing immobilized antigens, were washed twice with Tween–PBS and 0.1 mL of a test solution was transferred from the mixing plate to each well on the test plate. After incubation for 30 min at 30 °C, the test plate was washed three times with Tween–PBS. Then, 0.1 mL of GAMP, at a 1:2000 dilution in BSA-PBS, was added. The test plate was incubated for 30 min at 30 °C and then washed four times. An O-phenylenediamine (OPD) substrate solution [45] was added and incubated for 5 min at the ambient temperature. The reaction was stopped by adding 0.1 mL of 1 N hydrochloric acid and the absorbance at 490 nm was measured with an NEO microplate reader (Bio-Tek, Winooski, VT, USA). 

Nine selected monoclonal cell lines were used to produce ascites fluid in mice. The ascites fluid was partially purified by ammonium sulfate precipitation [34]; dialyzed against 0.1 M PBS, pH 7.2; and then lyophilized. Protein content of each of the preparations was determined using the BCA Protein Assay (Thermo Fisher, Pittsburgh, PA, USA). Following the purification of the mAbs, optimal concentrations of the immobilized antigen and each of the antibodies were determined. In the optimized immunoassays, mAb dilutions were made in PBS containing 1% BSA, which was added to stabilize the antibodies. For clones 1–4, the optimal mAb concentration was 50 ng/mL, while for clones 1–8 and 1–13, the optimal mAb concentrations were 200 ng/mL. For all three mAbs, the optimal concentration of NX3-BSA was 5 ng/mL. The optimized reagent concentrations were used in assays to evaluate solvent effects, cross-reactivity, and toxin recoveries from wheat.

### 5.5. Recovery of NX-2 and NX-3 from Wheat

Hard red winter wheat, hard white wheat, and soft white wheat were purchased in November 2022 from Palouse Trading (Palouse, WA, USA) and stored dry at 4 °C until use. Equal portions, by weight, of each variety were pooled and ground to the consistency of flour using a WonderMill (Reno, NV, USA). The pooled material was tested by LC-UV-MS and found to contain less than 10 μg/kg of DON, NX-2, and NX-3. For spiking of wheat with NX-2, a series of stock solutions were prepared in ACN at 1.875, 3.75, 5, 10, 20, and 37.5 μg/mL. For spiking of wheat with NX-3, a series was prepared in ACN at 12.5, 25, 75, 150, 400, and 1000 μg/mL. For spiking, 100 μL of a stock solution was added to 25 g of ground wheat, shaken briefly, and stored open to the air overnight at the ambient temperature to allow the evaporation of the ACN. This resulted in wheat spiked over the range of 7.5 to 150 μg/kg of NX-2 or 50 to 4000 μg/kg of NX-3. Quadruplicate samples were spiked and extracted at each toxin concentration (e.g., 24 spiked samples for each toxin). Each spiked sample was extracted once, and each extract was assayed with a minimum of 8 replicate wells.

Sodium chloride (2.5 g) was added to 25 g of ground wheat, followed by 100 mL of MeOH/H_2_O (80%, or 4 + 1 *v*/*v*). The mixture was shaken on a wrist-action shaker (Burrell Corporation, Pittsburg, PA, USA) for 30 min. The mixture was filtered through a Whatman 2V filter (Cytiva, Buckinghamshire, UK). One milliliter of the filtered extract was diluted with 7.0 mL of PBS and mixed gently. The diluted extracts, containing the equivalent of 0.03125 g of wheat per mL of extract, were tested without further dilution. For experiments to determine the impact of the wheat matrix on the performance of the ELISAs, the diluted matrix was prepared from control (un-spiked) wheat and used as the diluent to prepare calibration standards of NX-2 or NX-3.

## Figures and Tables

**Table 1:** Effects of methanol on the response of three NX antibodies to NX-2.

Methanol Conc.	Average IC_50_ ± S.D. (ng/mL) ^1^			Relative Increase in IC_50_^2^		
	mAb 1-4	mAb 1-8	mAb 1-13	mAb 1-4	mAb 1-8	mAb 1-13
0 (PBS alone)	0.40 ± 0.05	0.60 ± 0.14	0.55 ± 0.15	NA ^3^	NA	NA
10%	0.89 ± 0.04	1.10 ± 0.26	1.06 ± 0.16	225%	182%	193%
20%	1.93 ± 0.23	2.37 ± 0.61	2.39 ± 0.15	487%	392%	436%
30%	6.18 ±1.20	4.99 ± 0.91	6.76 ± 0.57	1560%	826%	1230%

^1^ Concentration of NX-2 to cause 50% inhibition of color development ± 1 standard deviation (n = 3 plates each). ^2^ Increase in IC_50_ due to MeOH, calculated as the IC_50_ indicated buffer divided by the IC_50_ in PBS. ^3^ NA: not applicable.

**Table 2:** Cross-reactivity of three NX mAbs in 10% MeOH/PBS.

Toxin	Average IC_50_ ± S.D. (ng/mL)			Cross-Reaction ^3^		
	mAb 1-4	mAb 1-8	mAb 1-13	mAb 1-4	mAb 1-8	mAb 1-13
NX-2	0.835 ± 0.111	0.890 ± 0.067	1.06 ± 0.05	100% (100%)	100% (100%)	100% (100%)
NX-3	11.8 ± 1.5	7.4 ± 0.8	8.5 ± 1.0	7.1% (6.2%)	12.0% (10.5%)	12.5% (10.9%)
DON	>500	>500	>500	<0.2% (<0.15%)	<0.2% (<0.16%)	<0.2% (<0.19%)
3-ADON	185 ± 44	155 ± 18	223 ± 8	0.45% <0.47%)	0.58% (<0.60%)	0.48% (<0.50%)
15-ADON	>500	>500	>500	<0.2% (<0.17%)	<0.2% (<0.19%)	<0.2% (<0.22%)
3,7 di-OH trichothecene ^1^	>500	>500	>500	<0.2% (<0.14%)	<0.2% (<0.15%)	<0.2% (<0.17%)
3-Ac, 7-OH trichothecene ^2^	>500	>500	>500	<0.2% (<0.16%)	<0.2% (<0.17%)	<0.2% (<0.20%)
7-OH diacetoxyscirpenol	>500	>500	>500	<0.2% (<0.19%)	<0.2% (<0.20%)	<0.2% (<0.24%)
T-2 toxin	>500	>500	>500	<0.2% (<0.24%)	<0.2% (<0.26%)	<0.2% (<0.31%)

^1^ Also known as 7-hydroxy isotrichodermol, it is equivalent to 15-deoxy-NX-3. ^2^ Also known as 7-hydroxy isotrichodermin, it is equivalent to 15-deoxy-NX-2. ^3^ Cross-reactivity on a mass concentration basis (molar basis in parentheses).

**Table 3:** Limits of detection and quantification and dynamic range for the mAb 1-8 ELISA.

Toxin	Test Mileu	LOD (ng/mL)	LOQ (ng/mL)	Dynamic Range (ng/mL)
NX-2	10% MeOH/PBS	0.11	0.23	0.23–3.61
	Diluted wheat matrix	0.08	0.24	0.24–3.97
NX-3	10% MeOH/PBS	0.32	1.48	1.48–44.9
	Diluted wheat matrix	0.66	1.85	1.85–48.1

**Table 4:** Recovery of NX-3 from spiked wheat.

NX-3 Added (μg/kg)	Average Recovery (%)	RSD ^1^ (%)
50 ^2^	100.5	15.7
100	95.0	5.9
300	99.1	10.7
600	102.2	10.7
1600	105.0	10.1
4000 ^3^	93.9	16.1

^1^ Relative standard deviation from four replicate samples of spiked wheat, analyzed and tested on separate days. ^2^ This level was selected to be near the LOQ of the method. ^3^ This level was above the dynamic range of the method.

**Table 5:** Recovery of NX-2 from spiked wheat.

NX-2 Added (μg/kg)	Average Recovery (%)	RSD ^1^ (%)
7.5 ^2^	81.1	8.0
15	77.2	12.0
20	79.2	9.5
40	86.5	7.5
80	92.5	5.7
150	90.2	10.0

^1^ Relative standard deviation from four replicate samples of spiked wheat, analyzed and tested on separate days. ^2^ This level was selected to be near the LOQ of the method.

## Data Availability

Data is contained within the article.

## Appendix A

**Table A1:** Effects of methanol on the signals from three NX mAbs in the absence of toxin.

Methanol Conc.	Average Absorbance ± S.D. ^1^		
	mAb 1–4	mAb 1–8	mAb 1–13
0 (PBS alone)	0.896 ± 0.112	0.962 ±0.196	1.006 ±0.149
10%	0.933 ± 0.087	0.953 ± 0.176	0.967 ± 0.209
20%	1.043 ± 0.138	1.005 ± 0.200	1.043 ± 0.247
30%	1.285 ± 0.168	1.157 ± 0.213	1.245 ± 0.220

^1^ Plus or minus one standard deviation.
